# Effects of biochar in combination with varied N inputs on grain yield, N uptake, NH_3_ volatilization, and N_2_O emission in paddy soil

**DOI:** 10.3389/fmicb.2023.1174805

**Published:** 2023-05-12

**Authors:** Zhenghua Yi, Paramsothy Jeyakumar, Chengcheng Yin, Haijun Sun

**Affiliations:** ^1^Co-Innovation Center for Sustainable Forestry in Southern China, College of Forestry, Nanjing Forestry University, Nanjing, China; ^2^Environmental Sciences, School of Agriculture and Environment, Massey University, Palmerston North, New Zealand; ^3^Key Laboratory of Soil and Water Conservation and Ecological Restoration of Jiangsu Province, Nanjing Forestry University, Nanjing, China

**Keywords:** biochar, N fertilizer, rice yield, NH_3_ volatilization, N_2_O emission

## Abstract

Biochar application can improve crop yield, reduce ammonia (NH_3_) volatilization and nitrous oxide (N_2_O) emission from farmland. We here conducted a pot experiment to compare the effects of biochar application on rice yield, nitrogen (N) uptake, NH_3_ and N_2_O losses in paddy soil with low, medium, and high N inputs at 160 kg/ha, 200 kg/ha and 240 kg/ha, respectively. The results showed that: (1) Biochar significantly increased the rice grain yield at medium (200 kg/ha) and high (240 kg/ha) N inputs by 56.4 and 70.5%, respectively. The way to increase yield was to increase the rice N uptake, rice panicle number per pot and 1,000 grain weight by 78.5–96.5%, 6–16% and 4.4–6.1%, respectively; (2) Under low (160 kg/ha) N input, adding biochar effectively reduced the NH_3_ volatilization by 31.6% in rice season. The decreases of pH value and NH_4_^+^-N content in surface water, and the increases of the abundance of NH_4_^+^-N oxidizing archaea and bacteria (AOA and AOB) communities contributed to the reduction of NH_3_ volatilization following the biochar application; (3) Under same N input levels, the total N_2_O emission in rice season decreased by 43.3–73.9% after biochar addition. The decreases of *nir*K and *nir*S gene abundances but the increases of *nos*Z gene abundance are the main mechanisms for biochar application to reduce N_2_O emissions. Based on the results of the current study, adding biochar at medium (200 kg/ha) N level (N200 + BC) is the best treatment to synchronically reduce NH_3_ and N_2_O losses, improve grain yield, and reduce fertilizer application in rice production system.

## 1. Introduction

China is a large rice growing country, with about 3.10 × 10^7^ ha of rice field cultivation area. China rice production accounts for 27% of global rice production and about 35% of China’s total grain production (Ullah et al., 2023). To meet the global population in rice demand, farmers apply inorganic nitrogen (N) fertilizer to increase rice yield (Chen C.R. et al., 2013; Chen T.T. et al., 2013; Chen et al., 2014). However, farmers often apply excessive N fertilizer inputs (more than 300–350 kg/ha/year) in the pursuit of maximum rice yields (Ali et al., 2020). Previous studies have demonstrated that approximately 50% of applied inorganic fertilizer N is lost either through emissions or leaching, which have detrimental effects to the atmosphere and water environment (Cao et al., 2013; Li et al., 2017). Of which, ammonia (NH_3_) volatilization is one major N loss pathway and account for 10–60% of the total N fertilizer input in the rice season. Moreover, nitrous oxide (N_2_O) emissions from rice fields can account for 7–11% of N_2_O emissions from agricultural fields in China (Zou et al., 2007; Cai et al., 2017). The N_2_O emission can deplete the stratosphere and accelerate climate change. Therefore, ensuring food security while reducing fertilizer N environmental losses by coupling other soil additives is a major ongoing concern in terms of agricultural production and ecological environment in China.

In previous literatures, several management practices have been recommended to reduce N losses in paddy fields such as the use of biochar. Biochar is a type of solid material produced by high-temperature pyrolysis carbonization of biomass under anaerobic conditions. It is characterized with well-developed pore structure, high carbon content and large specific surface area, which results to it having high stability and strong adsorption performance (Zou et al., 2007; Tian et al., 2020). Biochar itself carries micronutrients for crop growth (Pyoungchung et al., 2011) and can also increase soil carbon stocks, promote nutrient cycling and sequestration, and improve crop yields (Gaskin et al., 2008; Novak et al., 2009; Ali et al., 2020). For example, Mcconnell et al. (2007), demonstrated that biochar application with additional N (240 kg/ha) fertilizer increased rice yield by 49.7%, attributing to the increasing effective spike number, spike grain number and 1,000 grain weight. In addition, biochar application in agricultural fields can improve soil quality and reduce nutrient losses, thus increasing crop yields (Steiner et al., 2007). Apart from influencing yields, biochar application has been widely used to reduce pollutants in agricultural production processes (Sun et al., 2019). The amended biochar itself can adsorb NH_3_ and achieve NH_3_ volatilization reduction by enhancing soil N fixation capacity and increasing soil nitrification rate (Chen C.R. et al., 2013; Chen T.T. et al., 2013). However, the presence of high salt-based ions such as calcium and magnesium in biochar has been shown to have the potential to exchange with hydrogen and aluminum ions in the soil, etc., and this effect usually raises soil pH and leads to increased NH_3_ volatilization (Wang et al., 2011; Clough et al., 2013). Meanwhile, the effect of biochar on N_2_O emissions from rice fields has been inconsistently reported. Soil NH_4_^+^ content is a key factor affecting N_2_O emission rate, and biochar addition can slow down the denitrification process and reduce soil NH_4_^+^ content, thus reducing the N_2_O emission rate (Wang et al., 2011; Yang et al., 2020). However, some studies have also found no effect of biochar on soil N_2_O emission, or even a promoting effect (Clough et al., 2010; Wang et al., 2011). Several studies have demonstrated that the effects of biochar addition on NH_3_ volatilization and N_2_O emissions is highly influenced by environmental factors, biochar application rate, soil type and cropping system (Sha et al., 2019). However, limited studies investigated the comprehensive effects of biochar on NH_3_ volatilization and N_2_O emission in a whole crop grow cycle. Therefore, there is a need to undertake studies that cover a major share of the gaseous N loss pathways in rice production.

The integrated evaluation of crop yield, soil NH_3_ volatilization and N_2_O emission in response to N fertilizer application and exogenous substance addition has been a key area of research in the evaluation of the effects of on-farm management practices. However, most of the published recent studies have evaluated the effect of biochar application to agricultural fields based on a single level of N application rate (Ma et al., 2013). However, there is no clear evidence on the application of biochar with different rates of N fertilizer. This could help to underpin the best combination of biochar and N fertilizer with less N losses and without reducing rice yields. Hence, this study evaluated the effects of biochar addition on rice yield, NH_3_ volatilization and N_2_O emission at different levels of N supply. Further, explain the mechanistic effect of biochar on N losses based on N uptake, field water pH and ammonium N (NH_4_^+^-N), soil NH_4_^+^-N, and nitrate (NO_3_^−^-N) contents and functional microbial gene abundance by a pot experiment. The results and conclusions of the study can provide technical support and theoretical basis for the mutual interaction of N fertilizer and biochar to achieve N fertilizer reduction and biochar resource conservation, crop yield stabilization and environmental protection in rice production.

## 2. Materials and methods

### 2.1. Background information and soil column set-up

Test soil for this study was collected at depth of 0–20 cm from an approximately 30 ha paddy field in Zhoutie town of Yixing city (31° 28′ N, 119° 59′ E), Jiangsu Province, which is located at the Taihu Lake region of China. This region has a subtropical monsoon climate, with an annual mean air temperature and rainfall of 15.7°C and 1,177 mm, respectively. The soil was mixed, air-dried for approximately 10 days, ground and sieved through a 2-mm nylon sieve, and repacked layer-wise (0–10 and 10–20 cm) into soil columns (inner diameter 35 cm, height 28 cm) at similar bulk density (1.3 g/cm^3^) as in the field. About 20 kg soil was filled into each soil column according to the volume of soil column and the soil bulk density. The selected properties of 0–20 cm topsoil was as follows: pH 6.38 (soil: water ratio 1: 5), soil organic matter 29.2 g/kg, total N 1.72 g/kg, available P 23.1 mg/kg, available K 159.3 mg/kg, and CEC 22.6 cmol/kg. In this experiment, biochar was produced using wheat straw that had been heated to 500°C. The reactor was heated by a stepwise procedure under oxygen-limited conditions. For pyrolysis, the temperature was raised to 500°C at a rate of 5°C/min and held constant for 8 h. The measured properties of the biochar were pH 9.80, total N 0.81%, total C 37.5%, and BET surface area 32.0 m^2^/g. At the initiation of the experiment, at the same time of repacking soil into column/pot, biochar was mixed with the top layer (0–20 cm) soil.

### 2.2. Experimental treatments and rice management

This current experiment constituted seven treatments; urea only at low, medium, and higher rates of N with 160, 200, and 240 kg/ha, named NN160N200, andN240, respectively; N160, N200, and N240 plus biochar at 5 t/ha, named N160 + BC, N200 + BC, and N240 + BC, respectively; Meanwhile a and control treatment without urea and biochar was tested. The N fertilizer rates used on this study were based on deficiency, sufficiency, and over application. Each treatment was replicated three times, therefore there were totally 21 soil columns, Biochar was evenly incorporated into each soil column into the soil at a depth of 15 cm at the initiation of the experiment.

Rice (*Oryza sativa* L., var. Nangeng 46) seedlings (28 days old) were transplanted into the soil columns (three holes in each column and two plants per hole) on July 1, 2021. The total fertilizer N application was split into a basal dressing at transplanting, and two top-dressing during the season in the ratio of 40–30–30%, respectively. Application periods were July 1, July 16, and August 14 in 20,218. Calcium superphosphate and potassium chloride were applied to all treatments including the CK at the rates of 90 kg/ha (P_2_O_5_) and 150 kg/ha (K_2_O), respectively, at the time of transplanting as basal fertilizer. The floodwater was drained at a mid-season drainage period from July 31 to August 7, 2021 to control invalid tillering and improve the rice root development. During other times, a 3–5 cm depth floodwater level was maintained with tap water. Weeds and pests were controlled according to the local farmers’ traditional practices. The rice shoots (including straw and grain) were manually harvested on November 8, 2021.

### 2.3. Sampling and measurements

#### 2.3.1. Crop

Rice plants were harvested at the physiological maturity stage to determine grain yield and its components. Before harvesting, the plant height was measured using a ruler, and the yield-related agronomic traits (number of panicles, number of grains per panicle, and 1,000 seed weight) were also recorded. Rice straw and grain were oven-dried at 105°C for 30 min, and then dried at 80°C until constant weight. The dried plant samples were ground into powder using a high-speed crusher (DS-YM-001), passed through a 0.2 mm nylon sieve. Ground plant samples were kept in sealed containers until digestion. Sub-sample (0.25 g) of ground plant samples were digested in a mixture of H_2_SO_4_ and H_2_O_2_ and used for determination of total N content using the Kjeldahl method detailed in Sun et al. (2015). Rice NUE was calculated using Equation 1 outlined by Dong et al. (2015):

$\mathrm{NUE}\left(\%\right)=\frac{N_{F}-N_{0}}{N}\times 100\%$ (1)

Where $N_{F}$ and $N_{0}$ denote the N uptake as measured at harvest in the fertilizer applied and the control treatments (kg/ha), respectively, while N denotes the N fertilizer added rate (160, 200, and 240 kg/ha in the current work).

To reflect the leaf chlorophyll content, SPAD values of rice leaves were measured using the chlorophyll content meter (SPAD-502Plus, Japan) at the tillering, earing, and maturation stage, respectively. This was done by selecting three rice plants from each soil column and three leaves of each rice plant were measured. Therefore, the SPAD values presented in this study represented average SPAD value of the three plants in each replicate (Li et al., 2019).

#### 2.3.2. NH_3_ volatilization

The daily NH_3_ volatilization rates were measured at three N fertilizer applications, using the sponge absorption method (Rochette et al., 2013). The gas-capturing device was made of polyvinylchloride (PVC) plastic tube with an inner diameter of 15 cm and a height of 15 cm. Two sponges with a thickness of 2 cm and a diameter of 16 cm were dipped in 15 mL of phosphoglycerol (50 mL of phosphoric acid plus 40 mL of glycerol and then diluted to 1,000 mL with deionized water) and placed in a plastic tube. The lower sponge was 5 cm from the bottom of the tube and the upper sponge was at the top of the tube.

During sampling, the lower sponge was taken out (8: 00 am), immediately sealed in a bag, and replaced in the gas-capturing device with a new sponge also beforehand dipped in phosphoglycerol. The upper sponge was replaced once every 2 days. The sampled sponge was placed into a 500 mL plastic bottle, submerged in 300 mL of 1 M KCl, and shook at 180 r per minute for 1 h. The NH_4_^+^-N concentration in the extract was determined by an autoanalyzer (SKALAR San^++^ System, Netherlands). The NH_3_ volatilization was calculated using Equation 2 outlined below:

*ω =* $\frac{m\times V_{m}\times V_{e}}{V_{s}}$ ×10^−3^(2)

where, *ω*: NH_3_ content in a single collection device (mg); m : NH_4_^+^-N concentration (mg/L); $V_{m}$ : the volume of solution used to measure absorbance after constant volume (mL); $V_{e}$ : the KCl solution volume for extracting ammonium from sponge (mL); $V_{s}$ : the volume of extracting solution used for measurement (mL). The NH_3_ emission factor and yield-scale NH_3_ volatilization were calculated according to that introduced in our previous work (Min et al., 2021).

#### 2.3.3. N_2_O emission

The gas samples for N_2_O determination were collected using the modified closed chamber method as described in Min et al. (2021). The chamber was a transparent Plexiglas cylinder with 100 cm height and 36 cm inner diameter (adjusted for the height of rice plant and the pot size), and covered with Al foil to exclude light. It was fitted into a grove at the bottom (for sealing by tap water in the grove) and had a small fan at the top to properly mix gas before sampling.

Gas samples were collected using a plastic syringe at 15 min intervals. We took the gas samples on the 2nd, 4th, 6th, and 8th day after each N fertilization application and during water drainage period. Thereafter, sampling was done every 10 days until harvest. The sponge absorption device was temporarily moved out during the N_2_O measurement, to avoid any disturbance that may occur. When calculated the NH_3_ flux, we adjusted the cover time according to the fact. Gas sample collection was done between 6:00–8:00 a.m. Meanwhile, air temperature in each collection device was recorded at collection. Four gas samples were collected using a 50-mL medical syringe at 0, 15, 30, and 45 min after the collection device was sealed. The gas samples were then injected into pre-evacuated 50 ml vacuum bottles fitted with butyl rubber lids for laboratory analysis. The N_2_O concentrations were determined using a gas chromatograph (Agilent 7890B, Agilent Technologies, United States) at 350°C equipped with an electron capture detector (ECD). Total N_2_O emission was calculated from the individual fluxes and the interval times (Sun et al., 2022).

#### 2.3.4. NH_4_^+^-N, NO_3_^−^-N concentrations and pH in overlying water

Overlying water samples were collected using a syringe on the same day and time as NH_3_ volatilization samples collection. Collected water samples were filtered through a 0.45 μm membrane, then analyzed for pH using a combined reference electrode (Ф255 pH/temp/mV meter, Coulter Bechman Co., United States). A sub-sample of 50 ml filtered water was stored in clean plastic bottles at −20°C for further analysis. The concentrations of NH_4_^+^-N and NO_3_^−^-N in overlying water were determined by an autoanalyzer (SKALAR San^++^ System, Netherlands).

#### 2.3.5. Soil properties

At the end of the experimental period after rice harvest, three soil cores in each pot were randomly sampled at 0–20 cm depth using a soil drill (50 mm in diameter), top layer soil was sampled at selected points. Soil samples were composited, mixed manually, placed in self-sealing bags, and brought back to the laboratory in collar box with ice. Soil samples were divided into two parts: one was stored at −80°C for molecular analysis and another at −20°C for analysis of other properties. The soil pH was measured in the 1:2.5 (w/v) soil: water suspension using a combined reference electrode (Ф255 pH/temp/mV meter). Soil samples were extracted with 2.0 M KCl (1:5 soil: extractant, w/v) for NH_4_^+^-N and NO_3_^−^-N determination. Soil extracts were filtered through a 0.45 μm membrane filter and the NH_4_^+^-N and NO_3_^−^-N concentrations were determined using an autoanalyzer (SKALAR San^++^ System, Netherlands). The gene copy numbers of AOA and AOB *amo*A, *nir*K, *nir*S, and *nos*Z of soil samples were determined by Shanghai Majorbio Biomedical Co., Ltd. according to the procedures detailed in Chu et al. (2020) and Ye et al. (2021).

### 2.4. Data analysis

A statistical analysis was performed using SPSS 22.0, and one-way analysis of variance (ANOVA) was used to determine the significance of the difference between treatments. The level of significance was measured using Duncan’s multiple-comparison test (*p* < 0.05).

## 3. Results

### 3.1. Rice yield and nitrogen uptake

#### 3.1.1. Rice yield and its component factors

The results in Table 1 showed that N200 and N240 treatments significantly (*p* < 0.05) increased the straw biomass by 54.0 and 77.0% relative to the control treatment. Both N160 and N160 + BC had no difference in straw biomass compared to the control. The combinations of BC with N200 and N240 significantly (*p* < 0.05) increased rice straw biomass by 79.5 and 42.3% compared to N200 and N240 alone, respectively. However, there was no difference in straw biomass between N160 + BC and N160. Interestingly, the grain yield in N added treatments (70.53–145.50 g/pot) were significantly (*p* < 0.05) higher than the no N added control treatment (42 g/pot). Moreover, N200 + BC and N240 + BC treatments significantly (*p* < 0.05) promoted the rice grain yield by 31.9 and 70.6% relative to N200 and N240 treatments, respectively. Nevertheless, amendment of biochar induced no difference in rice grain yield at low N input level (160 kg/ha).

**Table 1:** Effects of biochar application and N fertilizer reduction on rice straw biomass, grain yield and its component factors.

Treatment	Straw biomass (g/pot)	Grain yield (g/pot)	Yield component factors		
			Panicle number	Grain number per panicle	Thousand seed weight (g)
Control	96.03 ± 24.47 d	42.63 ± 4.64 d	16.00 ± 2.00 f	109.00 ± 14.00 b	24.73 ± 0.58 ab
N160	115.50 ± 6.71 cd	72.47 ± 1.75 c	29.00 ± 4.00 de	117.00 ± 11.00 ab	23.93 ± 0.42 b
N160 + BC	118.55 ± 6.77 cd	70.53 ± 1.96 c	27.00 ± 3.00 e	120.00 ± 10.00 ab	24.27 ± 0.50 b
N200	147.88 ± 13.11 bc	93.03 ± 19.6 b	32.00 ± 3.00 cd	119.00 ± 20.00 ab	24.00 ± 0.20 b
N200 + BC	265.44 ± 14.02 a	145.50 ± 3.00 a	48.00 ± 3.00 a	128.00 ± 20.00 ab	25.47 ± 0.23 a
N240	169.97 ± 14.93 b	82.20 ± 2.00 bc	35.00 ± 2.00 c	103.00 ± 11.00 b	24.27 ± 0.50 b
N240 + BC	241.87 ± 30.72 a	140.23 ± 7.48 a	41.00 ± 3.00 b	140.00 ± 7.00 a	25.33 ± 0.70 a

Data in the table are mean ± standard deviation (*n* = 3); different lowercase letters in the same column indicate significant differences between treatments (*p* < 0.05).

Moreover, the number of panicles and 1,000 grain weight of rice in N200 + BC treatment were significantly (*p* < 0.05) 16 panicles/pot and 6.1% higher than N200, respectively. Compared with N240 treatment, however, N240 + BC treatment significantly (*p* < 0.05) increased the number of panicles, grains per panicle and 1,000 grain weight of rice by 6 panicles/pot, 35.9, and 4.4%, respectively (Table 1).

#### 3.1.2. Rice plant height and leaf SPAD

**Figure 1:**
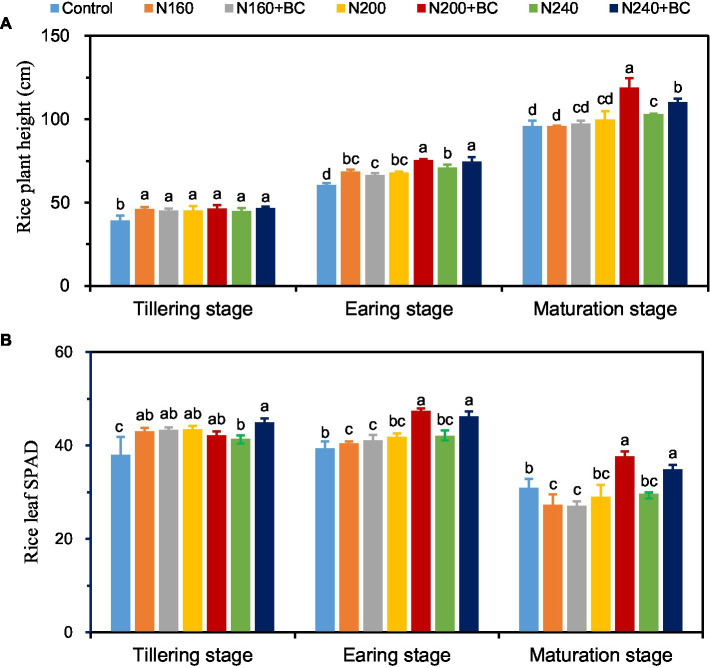
Effects of biochar application and N fertilizer reduction on rice plant height **(A)** and flag leaf SPAD **(B)** at different growth stages. Different lowercase letters above the columns indicate significant differences between treatments (*p* < 0.05).

At the tillering stage, either N fertilizer reduction or biochar addition had no effect on rice plant height (Figure 1A), but the SPAD value of rice leaf in N240 + BC treatment was significantly increased by 8.6% (*p* < 0.05) compared with N240 treatment (Figure 1B). At earing and maturation stages, rice plant height of rice was significantly (*p* < 0.05) increased by 5.0–11.0% and 7.1–19.5%, and rice leaf SPAD value was significantly (*p* < 0.05) increased by 9.9–13.4% and 17.6–29.9% (*p* < 0.05) when biochar was applied at medium (200 kg/ha) and high N (240 kg/ha) application levels. Among the different treatments at both early and maturing stage, the N200 + BC treatment had the highest values in terms of rice plant height and leaf SPAD value (Figure 1).

#### 3.1.3. N content and uptake of rice straw and seeds

No significant difference in N content and uptake by rice straw among the treatments with N fertilizer but without biochar (Table 2). However, the seed N content and uptake increased when N addition increased from 160 kg/ha to 200 kg/ha, but decreased when N addition further increased to 240 kg/ha The N200 treatment showed a significant (*p* < 0.05) increase of 9.7 and 40.7% in seed N content and uptake, compared with N160 treatment, respectively. Biochar addition significantly (*p* < 0.05) increased rice straw N content by 39.8–75.7% and N uptake by 148–152% at medium and high N supply. Overall, biochar addition exerted no effect on seed N content of rice receiving same N fertilizer, while biochar addition significantly (*p* < 0.05) increased seed N uptake by 54.7–73.0% at middle (200 kg/hm^2^) and high (240 kg/hm^2^) N supply. Meanwhile, the total N uptake (straw + seeds) in N200 + BC and N240 + BC treatments was 1.8 and 2.0 times higher compared to N200 and N240 treatments, respectively (Table 2).

**Table 2:** Effect of biochar addition and N fertilizer reduction on total N content and uptake of rice straw and grain.

	Nitrogen content (g/kg)		Nitrogen uptake (g/pot)		
Treatment	Rice straw	Rice grain	Rice straw	Rice grain	Total
Control	4.39 ± 0.01 c	18.20 ± 0.10 cd	0.36 ± 0.03 b	0.73 ± 0.03 d	1.09 ± 0.06 d
N160	4.36 ± 0.18 c	18.58 ± 0.36 bcd	0.50 ± 0.05 b	1.35 ± 0.03 c	1.85 ± 0.02 c
N160 + BC	4.54 ± 0.27 c	18.08 ± 0.54 d	0.54 ± 0.03 b	1.27 ± 0.02 c	1.81 ± 0.02 c
N200	4.40 ± 0.09 c	20.39 ± 0.68 a	0.65 ± 0.07 b	1.90 ± 0.46 b	2.56 ± 0.53 b
N200 + BC	6.15 ± 1.69 b	20.19 ± 0.21 a	1.64 ± 0.46 a	2.94 ± 0.09 a	4.57 ± 0.37 a
N240	4.15 ± 0.09 c	19.34 ± 1.25 abc	0.71 ± 0.22 b	1.59 ± 0.13 bc	2.30 ± 0.08 bc
N240 + BC	7.29 ± 0.08 a	19.63 ± 0.48 ab	1.76 ± 0.27 a	2.75 ± 0.18 a	4.52 ± 0.38 a

Data in the table are mean ± standard deviation (*n* = 3); different lowercase letters in the same column indicate significant differences between treatments (*p* < 0.05).

### 3.2. NH_3_ volatilization, emission factor and yield-scale NH_3_ volatilization

As shown in Supplementary Figure 1, during BF observation, the peak NH_3_ volatilization rate (24.95–67.03 mg/pot/d) was observed on the third days after BF, while the peak NH_3_ volatilization rate (29.48–110.63 mg/pot/d) was observed on the first day after SF2. Except for the N240 treatment, NH_3_ volatilization could reach its peak (41.33–74.84 mg/pot/d) within the first 3 days after SF1. The NH_3_ volatilization rate in all N fertilizer applied treatments dropped to a low level as that in the control treatment within 7 days after N each fertilizer application conducted.

The cumulative NH_3_ volatilizations in the rice season under N applied treatments were 0.20–0.53 g/pot, accounting for 10.5–28.7% of the fertilizer N input into the rice paddy. The NH_3_ losses after BF, SF1 and SF2 were 0.06–0.18 g/pot, 0.09–0.23 g/pot, and 0.05–0.20 g/pot, respectively (Table 3). N160 + BC treatment reduced the NH_3_ volatilization at both SF1 (38.9%) and SF2 (37.2%) observations, compared with N160 treatment, and this effect resulted in an overall significant (*p* < 0.05) reduction in cumulative amount of NH_3_ volatilization by 31.6%. Moreover, the application of biochar at N160 level significantly (*p* < 0.05) reduced the NH_3_ emission factor by 37.2%. Nevertheless, the total NH_3_ losses and emission factor in the rice season were not influenced by biochar at 200 kg/ha and 240 kg/ha applications. Interestingly, biochar addition significantly (*p* < 0.05) reduced yield-scale NH_3_ volatilization at all three N application levels by 29.7, 52.7, and 46.3% relative to N160, N200, and N240 kg/ha supply, respectively (Table 3).

**Table 3:** Effect of biochar application and N fertilizer reduction on the accumulation of NH_3_ volatilization, emission factor and yield-scale NH_3_ volatilization at different fertilization periods and the whole reproductive period of rice.

Treatment	NH_3_ volatilizations (g/pot)				Emission factor	Yield-scale NH_3_ volatilization
	BF	SF1	SF2	Accumulation	%	g/kg
Control	0.02 ± 0.00 d	0.01 ± 0.00 d	0.03 ± 0.01 f	0.06 ± 0.01 d	–	1.31 ± 0.09 d
N160	0.06 ± 0.01 c	0.18 ± 0.08 ab	0.14 ± 0.01 b	0.38 ± 0.09 b	28.7 ± 7.6 a	5.25 ± 1.22 b
N160 + BC	0.06 ± 0.02 c	0.11 ± 0.01 c	0.09 ± 0.01 c	0.26 ± 0.01 c	18.0 ± 1.0 b	3.69 ± 0.24 c
N200	0.07 ± 0.02 c	0.13 ± 0.04 bc	0.06 ± 0.02 de	0.27 ± 0.03 c	15.0 ± 2.1 bc	2.98 ± 0.81 c
N200 + BC	0.06 ± 0.02 c	0.09 ± 0.03 c	0.05 ± 0.01 ef	0.20 ± 0.04 c	10.5 ± 3.0 c	1.41 ± 0.30 d
N240	0.15 ± 0.02 b	0.18 ± 0.03 ab	0.20 ± 0.02 a	0.53 ± 0.02 a	28.1 ± 1.0 a	6.47 ± 0.81 a
N240 + BC	0.18 ± 0.00 a	0.23 ± 0.02 a	0.07 ± 0.2 cd	0.49 ± 0.02 a	25.3 ± 1.2 a	3.47 ± 0.08 c

BF, basal fertilizer; SF1, first supplementary; SF2, second supplementary fertilization of urea-N in rice season. Data are means ± SD (*n* = 3); different lowercase letters in the same column indicate significant differences between treatments (*p* < 0.05).

### 3.3. N_2_O emission

**Figure 2:**
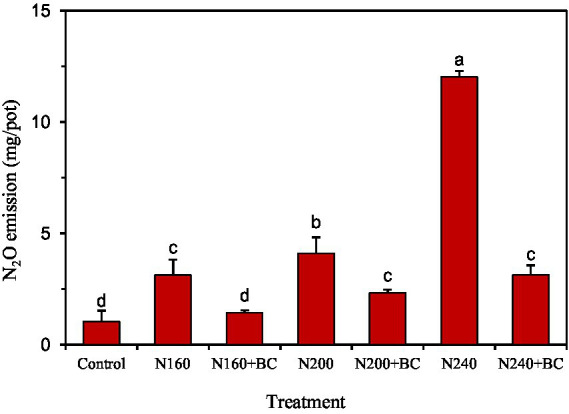
Effects of biochar application and N fertilizer reduction on cumulative N_2_O emission from rice paddy. Different lowercase letters above the columns indicate significant differences between treatments (*p* < 0.05).

Under no biochar additions, N_2_O emissions in the rice season increased significantly (*p* < 0.05) with the increasing N application (Figure 2). Biochar addition significantly (*p* < 0.05) reduced the cumulative N_2_O emissions in rice season by 54.1, 43.3, and 73.9% relative to N160, N200, and N240, respectively. Meanwhile, results in Figure 2 show that the cumulative N_2_O emissions of N200 + BC and N240 + BC treatments in rice season can be reduced to the N160 level, and the N_2_O emissions of N160 + BC treatment can be reduced to the control treatment level.

### 3.4. NH_4_^+^-N and NO_3_^−^-N concentrations in overlying water

#### 3.4.1. NH_4_^+^-N concentration in overlying water

**Figure 3:**
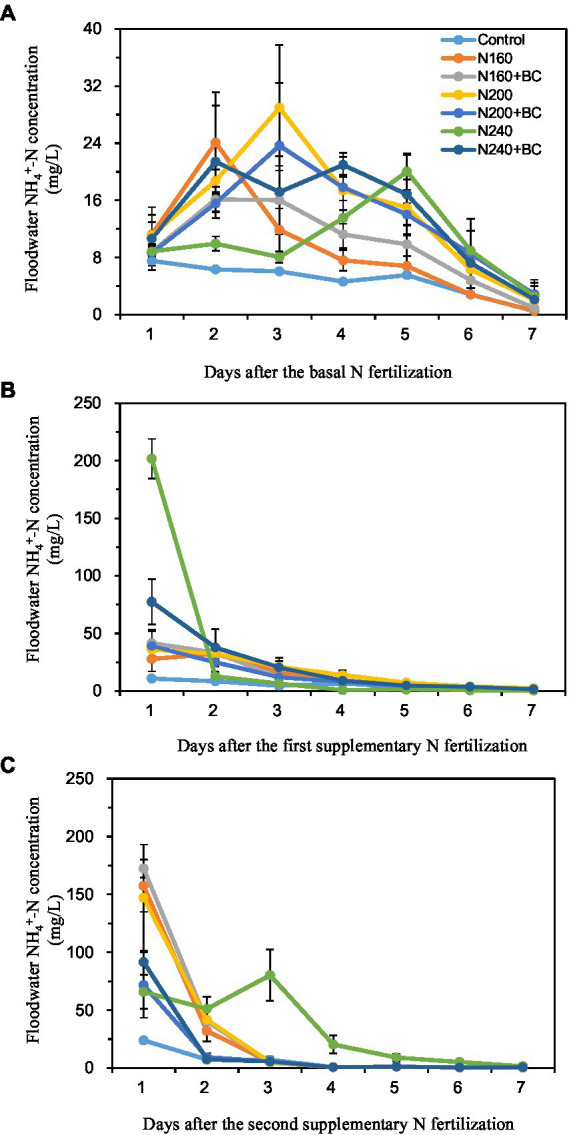
Effect of biochar application and N fertilizer reduction on ammonium nitrogen (NH_4_^+^-N) concentration in overlying water after applications of basal fertilizer (BF, **A**), first supplementary fertilizer (SF1, **B**) and second supplementary fertilizer (SF2, **C**).

The peak NH_4_^+^-N concentrations in the overlying water of each treatment during the BF occurred on the 2–5 days (16.1–29.0 mg/L) after N fertilizer application (Figure 3A). Except for the N240 treatment, on the first day after SF1 and SF2 applied, the NH_4_^+^-N concentrations of overlying water came to the peak with 27.8–77.5 mg/L and 71.7–172.4 mg/L, respectively (Figures 3B,C). After reaching the peak, the NH_4_^+^-N concentration in the overlying water of each treatment decreased rapidly to no significant difference among all treatments (Figure 3).

At the BF observation, compared with N200, the average NH_4_^+^-N concentration of overlying water in N200 + BC decreased by 8.5%. However, biochar addition into low (160 kg/ha) and high (240 kg/ha) N supplied treatments increased the average NH_4_^+^-N concentration of overlying water by 4.4 and 33.9%, respectively. After the SF1 and SF2 applied, the addition of biochar at low (160 kg/ha) N supply level increased the average NH_4_^+^-N concentration of overlying water by 10.0 and 11.8%, respectively. Nevertheless, biochar addition at medium (200 kg/ha) and high (240 kg/ha) N supply levels reduced the mean overlying water NH_4_^+^-N concentrations by 20.3–54.5% and 31.0–53.7%, respectively, during the same periods.

#### 3.4.2. NO_3_^−^-N concentration in overlying water

**Figure 4:**
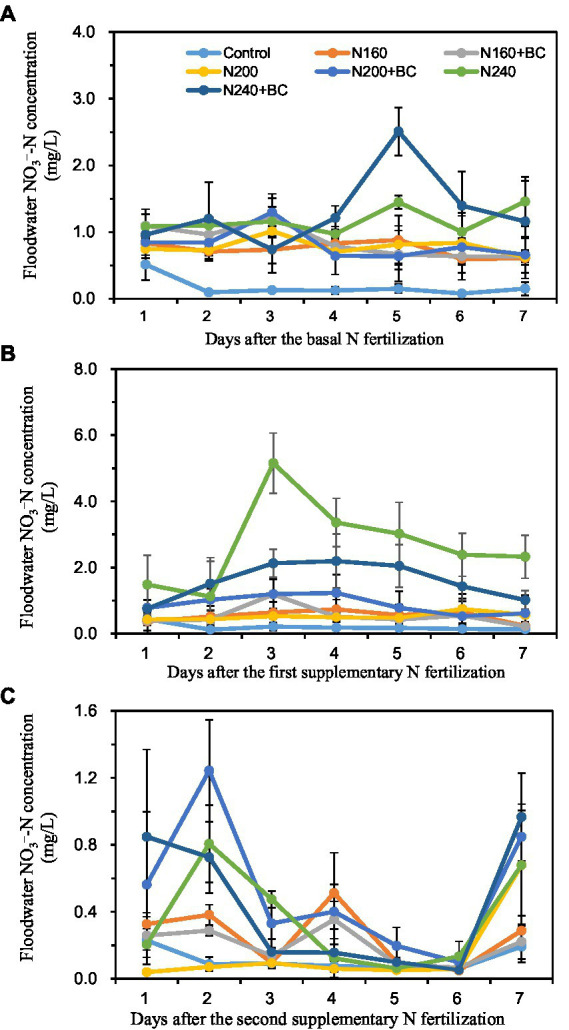
Effect of biochar application and N fertilizer reduction on nitrate N (NO_3_^−^-N) concentration of overlying water after applications of basal fertilizer (BF, **A**), first supplementary fertilizer (SF1, **B**) and second supplementary fertilizer (SF2, **C**).

During the BF, the addition of biochar at all three levels of N supply increased the mean NO_3_^−^-N concentration in the overlying water by 16.2, 3.8, and 11.0%, respectively (Figure 4A). During the SF1, the addition of biochar at the medium (200 kg/ha) N level increased the mean NO_3_^−^-N concentration in the overlying water by 66.0%, but at the high (240 kg/ha) N level it decreased the mean NO_3_^−^-N concentration in the overlying water by 41.3%. During the SF2, the average NO_3_^−^-N concentration in the overlying water of N200 + BC and N240 + BC treatments was 3.5 and 1.2 times higher than that of N200 and N240 treatments, respectively (Figures 4B,C).

### 3.5. Soil properties

#### 3.5.1. Topsoil NH_4_^+^-N and NO_3_^−^-N content

**Figure 5:**
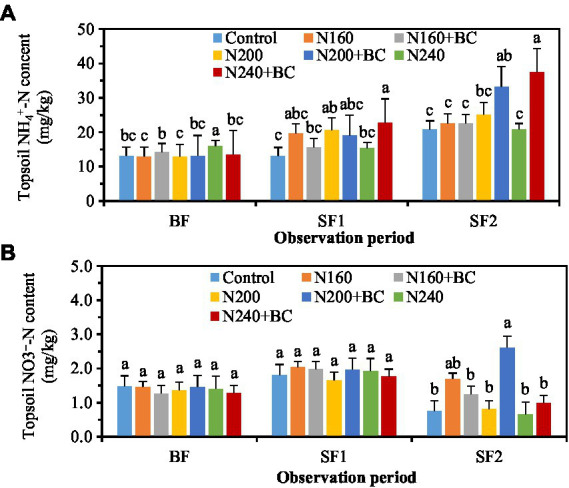
Effects of biochar addition and N reduction on the contents of NH_4_^+^-N **(A)** and NO_3_^−^-N **(B)** in soil at different fertilizer stages. The BF, SF1, and SF2 refer to the basal, first and second supplementary fertilizations, respectively. Different lowercase letters above the columns indicate significant differences between treatments (*p* < 0.05).

During the BF, biochar had varied effects on soil NH_4_^+^-N content under low (160 kg/ha) and high (240 kg/ha) N fertilizer application conditions. The soil NH_4_^+^-N content in N160 + BC treatment was significantly (*p* < 0.05) higher than N160 treatment by 9.8%, while that in N240 + BC was significantly (*p* < 0.05) lower than N240 treatment by 15.0% (Figure 5A). The soil NH_4_^+^-N contents in the N240 + BC treatment were significantly (*p* < 0.05) 48.8 and 79.7% higher than N240 treatment during the SF1 and SF2, respectively.

The soil NO_3_^−^-N content was not remarkably affected by N fertilizer application and biochar addition during the BF and SF1 (Figure 5B). During the SF2, biochar amendment at medium (200 kg/ha) N levels significantly (*p* < 0.05) increased the soil NO_3_^−^-N content, and the N200 + BC treatment had 3.3 times more soil NO_3_^−^-N content than the N200 treatment (Figure 5B).

#### 3.5.2. Community abundance of ammonia oxidizing and denitrifying bacteria

Table 4 shows that the number of functional genes of ammonia-oxidizing archaea (AOA) and ammonia-oxidizing bacteria (AOB) *amo*A in paddy soil ranged from 2.23–6.22 × 10^5^ copies/g and 1.73–5.09 × 10^5^ copies/g, respectively. And the number of functional genes of denitrifying bacteria *nir*S, *nir*K and *nos*Z ranged from 1.51–16.34 × 10^7^, 2.76–14.03 × 10^6^ copies/g and 0.33–3.83 × 10^6^ copies/g. For all treatments, the *nirS* gene showed higher dominance role. The community abundance of AOB and denitrifying bacteria showed a pattern of decreasing with increasing N application levels (Table 4). However, in N160 + BC and N240 + BC treatments, the soil AOA abundance significantly (*p* < 0.05) increased by 37 and 146%, relative to their counterparts N160 and N240 treatments, respectively. However, soil AOB abundance under medium (200 kg/ha) N supply conditions was significantly (*p* < 0.05) increased by 1.5 times with the addition of biochar.

**Table 4:** Effect of biochar application and nitrogen fertilizer reduction on the abundance of soil ammonia oxidizing and denitrifying bacteria communities.

	AOA	AOB	*nir*K	*ni* S	*nos*Z
Treatment	10^5^ copies/g	10^5^ copies/g	10^6^ copies/g	10^7^ copies/g	10^6^ copies/g
Control	2.4 ± 0.62 c	2.44 ± 4.49 c	8.73 ± 0.49 b	3.02 ± 0.09 d	0.59 ± 0.01 cd
N160	3.28 ± 0.64 c	3.74 ± 0.16 b	14.03 ± 1.88 a	12.21 ± 2.65 b	3.83 ± 0.85 a
N160 + BC	4.50 ± 9.37 b	3.84 ± 7.62 b	10.48 ± 0.88 b	2.69 ± 0.41 d	1.03 ± 0.05 cd
N200	2.23 ± 7.09 c	2.04 ± 4.99 d	3.16 ± 1.32 c	7.53 ± 1.39 c	1.37 ± 0.37 c
N200 + BC	3.32 ± 7.88 c	5.09 ± 0.42 a	5.02 ± 1.01 c	6.85 ± 2.11 c	1.26 ± 0.11 c
N240	2.53 ± 0.55 c	1.73 ± 5.54 d	2.76 ± 0.14 c	1.51 ± 0.15 d	0.33 ± 0.04 d
N240 + BC	6.22 ± 7.96 a	2.89 ± 3.37 cd	10.16 ± 2.69 b	16.34 ± 1.76 a	2.45 ± 0.80 b

Different lowercase letters in the same column indicate significant differences between treatments (*p* < 0.05).

N160 + BC had significantly (*p* < 0.05) lower number of *nir*S, *nir*K, and *nos*Z functional genes by 78.0, 25.3, and 73.1%, respectively compared to N160. In contrast, the copies of *nir*S, *nir*K, and *nos*Z in N240 + BC soil were 10.8, 3.7, and 7.4 times significantly (*p* < 0.05) higher than N240 soil. No significant effect of biochar on the copies of all three denitrifying bacterial functional genes was found at medium (200 kg/ha) N supply level.

## 4. Discussion

### 4.1. Effect and mechanism of biochar on rice yield

Both exogenous material addition and N fertilizer application played a crucial role in the formation of rice yield (Timsina et al., 2001; Zhang et al., 2020). The results of this study showed that the combinations of N200 + BC and N240 + BC were effective in improving rice yield (Table 1). Similar results have been confirmed by Feng et al. (2021). They demonstrated that application of N fertilizer at 240 kg/ha with biochar increased the rice yield by 9.3% in a pot experiment. Results presented in the current study demonstrated that the addition of biochar at medium (200 kg/ha) and high (240 kg/ha) N levels promoted rice grain yield through increasing effective panicle number and 1,000 seed weight. However, there was no significant difference in panicle number, grain number per panicle and 1,000 seed weight between N160 + BC and N160 treatments, explaining the no yield-increasing effect of biochar on rice at 160 kg/ha N addition. Meanwhile, it has been shown that biochar can increase the growth rate of seedling rice and its mineral element uptake and dry matter accumulation (Ray et al., 2012). Further, combination of biochar and N at 240 kg/ha increased the rice plant height and leaf SAPD values (Figure 1). Therefore, this effect can also be inferred that promoting dry matter accumulation in rice and increasing leaf SPAD values are also one of the mechanisms of biochar to increase crop yield.

In terms of nutrient uptake, biochar addition has been well reported to improve crop N fertilizer utilization (Van Zwieten et al., 2010; Huang et al., 2018). Similarly, in this experiment we found that the addition of biochar at N200 and N240 treatments increased rice seed N and total plant N uptake capacities (Table 2). Therefore, promoting N uptake and utilization of rice is the second mechanism of biochar to improve rice yield under 200–240 kg/ha N supply conditions. The increase in N uptake under these treatments can be associated with the effect of biochar increasing the activity of soil microorganisms by providing them with an unstable carbon source. The presence of a C source can promote soil N mineralization thus improving soil N availability, and promotes plant N uptake (Saifullah et al., 2018). In addition, the addition of biochar can improve the stability of soil aggregates and crop root architecture (especially increase the number of fine roots) (Backer et al., 2017), as well as promote the uptake of other fast-acting nutrients by rice, thus ensuring the nutrient supply for the whole growth cycle of rice.

### 4.2. Effect of biochar on soil NH_3_ volatilization under different N fertilizer levels

This study found that the effect of biochar on NH_3_ volatilization in rice season was related to N input level, with a significant reduction of NH_3_ volatilization only at low (160 kg/ha) N level (Table 3). Under different experimental conditions, different views on the impact of biochar addition on NH_3_ volatilization in rice field ecosystem including promotion, reduction, and no impact, were reported (Meng et al., 2019; Shaukat et al., 2019). Liu et al. (2020) reported that appropriate N reduction form 180 kg/ha to 150 kg/ha with biochar helped to reduce NH_3_ volatilization and these results are consistent with the findings of our present study. In addition to environmental factors such as wind speed and temperature, the main factors affecting NH_3_ volatilization in rice fields include soil and overlying water pH and NH_4_^+^-N concentration, especially the changes in the corresponding indicators within 1 week after N fertilizer application (Soares et al., 2012; Mandal et al., 2018). The reduction effects of biochar on NH_3_ volatilization under low (160 kg/ha) N condition in this experiment were mainly found the SF1 (39%) and SF2 (36%). As shown in the Supplementary Figure 2, the addition of biochar at low (160 kg/ha) N input at both the SF1and SF2 reduced the mean pH of overlying water by 0.05 units. In addition, the mean soil NH_4_^+^-N and pH decreased by 4.07 mg/kg and 0.07 units, respectively, during the SF1 (Figure 5A; Supplementary Figure 3). In this study, the reduction of pH value and NH_4_^+^-N content by the N160 + BC at the SF1 and SF2 is linked to the reduction of NH_4_^+^-N potential for conversion to NH_3_, thus inhibiting the volatilization of NH_3_ (Feng et al., 2022).

Further, the addition of biochar at low (160 kg/hm^2^) N levels can increase the community abundance of AOB (Table 4). This is because previous study has found that the surface structural properties of biochar could provide habitat for AOB and increase the abundance and activity of AOB, which is consistent with the results of this work (Cheng et al., 2012). Increased abundance of AOB communities enhances soil nitrification, which can improve soil utilization of NH_4_^+^-N and reduce NH_4_^+^-N concentration in the soil liquid phase, thus reducing NH_3_ volatilization from rice fields (He et al., 2019). The soil urease activity directly affects the urea hydrolysis process, and largely determines the soil NH_3_ emission rate and amount (Yi et al., 2021). Previous studies have shown that biochar can adsorb urease molecules, and then protect the binding sites of enzymatic reaction, thus to prevent the enzymatic reaction and reduce NH_3_ volatilization (Noyce et al., 2015). In this experiment, the addition of biochar at low (160 kg/ha) N level may inhibit soil urease activity and thus decreased NH_3_ volatilization from rice fields, but the exact effect needs to be further investigated. In addition, according to the suggestion of Nannipieri et al. (2019), more frequent soil sampling should be conducted, after the N fertilization, to reveal the enzymatic mechanism of biochar effects on the NH_3_ volatilization from paddy systems.

### 4.3. Effect of biochar on soil N_2_O emission under different N fertilizer levels

The results of this experiment showed that biochar addition under different N application conditions was effective in reducing N_2_O emissions in the rice season (Figure 2), and this finding is consistent with previous report (Fan et al., 2020). The effect of biochar on N_2_O emissions is associated with the ability of biochar to alter the conversion of soil N nutrients (NO_3_^−^-N and NH_4_^+^-N) (Harter et al., 2014; Liu et al., 2019). Nguyen et al. (2017) found that biochar addition reduced the effective source of N for soil nitrifying and denitrifying bacteria, which in turn contributed to the reduction of N_2_O emissions. In this study, soil NH_4_^+^-N and NO_3_^−^-N contents were reduced by 0.91 and 0.23 mg/kg, respectively, after biochar addition with N at 160 kg/ha (Figures 5A,B), which corresponded with nitrification and denitrification inhibited, thus reducing N_2_O emissions. In addition, pH changes are also the main controlling factor for differences in soil N_2_O emission between the treatments with or without biochar (Wang et al., 2018), especially when the nitrification processes dominate are reduced in soils with lower pH (Yoo et al., 2016). In this study, the average pH values of the soil following biochar addition at 200–240 kg/ha were reduced (Supplementary Figure 3), which also contributed to the reduction of soil N_2_O emissions by biochar addition at medium and high N inputs. Our results further demonstrated that under different N application levels, the mechanism of biochar to reduce N_2_O emission is varied.

Functional soil microbial communities represented by *nir*S, *nir*K, and *nos*Z genes play key roles in regulating N_2_O emissions (Wang et al., 2013; Harter et al., 2016). In this study, biochar application at low (160 kg/ha) N levels decreased soil *nir*S and *nir*K gene copy numbers, while biochar application at high (240 kg/ha) N levels increased soil *nos*Z gene copy numbers (Table 4). According to previous study, the reduction of N_2_O emissions as biochar was linked to the decreased *nir*S and *nir*K genes copies but the increased *nos*Z gene copies or a decrease in the ratio of (*nir*K + *nir*S)/*nos*Z in other word (Shi et al., 2019). Duan et al. (2018) found that biochar could increase the abundance of *nos*Z genes in soil, thus effectively reducing N_2_O emissions, like the results of this study. The addition of biochar at low (160 kg/ha) N level in this study inhibited soil autotrophic nitrification and denitrification processes by reducing soil *nir*S and *nir*K gene abundance, and at medium (200 kg/ha) N level by reducing soil *nir*S gene abundance. In contrast, the addition of biochar at high (240 kg/ha) N levels is effective in reducing N_2_O emissions by increasing the abundance of soil *nos*Z genes, increasing N_2_O reductase activity, and promoting the catalytic process of N_2_O reduction to N_2_.

## 5. Conclusion

The combined effects of biochar addition and N reduction on rice yield and N uptake, NH_3_ and N_2_O losses in paddy soil and the underlying mechanisms were evaluated by a soil column experiment. The main conclusions were:

(1) The yield increasing effect of biochar can be realized at medium (200 kg/ha) to high (240 kg/ha) N inputs, attributing to the improving N uptake and increasing panicle number, grain number per panicle and 1,000 seed weight. In particular, the highest yield was achieved by combination of biochar and N fertilizer at medium (200 kg/ha) level.

(2) Biochar addition only effectively reduced the NH_3_ volatilization in rice season with low (160 kg/ha) N supply condition. Interestingly, however, at equal N input level, yield-scale NH_3_ volatilizations were reduced by biochar addition.

(3) Biochar addition was effective in reducing the N_2_O emissions from rice paddy receiving inorganic N fertilizer from low (160 kg/ha) to high (240 kg/ha) inputs. Varied changes in the functional genes, including *nir*S, *nir*K, and *nos*Z, explained the inhibiting effects of biochar on the N_2_O emission at different N inputs.

(4) We recommend the addition of biochar at medium (200 kg/hm^2^) N levels, which can archive the synergistic benefits of reducing inorganic N fertilizer, promoting crop yield, and decreasing N environmental losses.

## Data availability statement

The raw data supporting the conclusions of this article will be made available by the authors, without undue reservation.

## Author contributions

ZY and CY conducted the experiment, obtained the data, and drafted the manuscript. PJ drafted and revised the manuscript. HS designed the experiment, provided the fund support, and drafted and revised the manuscript. All authors contributed to the article and approved the submitted version.

## Funding

This work was finically supported by the Science and Technology Innovation Program of Jiangsu Province, China for “Carbon Dioxide Emission Peaking and Carbon Neutrality” (BE2022307), the Jiangsu Agricultural Science and Technology Independent Innovation Fund [CX(21)3066], the National Natural Science Foundation of China (31972518), and the Qing Lan Project of Jiangsu Province of China.

## Conflict of interest

The authors declare that the research was conducted in the absence of any commercial or financial relationships that could be construed as a potential conflict of interest.

## Publisher’s note

All claims expressed in this article are solely those of the authors and do not necessarily represent those of their affiliated organizations, or those of the publisher, the editors and the reviewers. Any product that may be evaluated in this article, or claim that may be made by its manufacturer, is not guaranteed or endorsed by the publisher.

## Supplementary material

The Supplementary material for this article can be found online at: https://www.frontiersin.org/articles/10.3389/fmicb.2023.1174805/full#supplementary-material
