# Supplementary material for: Effects of biochar in combination with varied N inputs on grain yield, N uptake, NH3 volatilization, and N2O emission in paddy soil
Source: Front Microbiol. 2023 May 12;14:1174805. doi: 10.3389/fmicb.2023.1174805 (PMC10214156; doi:10.3389/fmicb.2023.1174805)
Supplement: Supplementary file 1 [file Data_Sheet_1.docx]

**Supplementary Figure 1**

**Supplementary Figure 1.** Effect of biochar application and N fertilizer reduction on the dynamics of soil NH_3_ volatilization during the observations after three fertilizer N applied.

**Supplementary Figure 2**

**Supplementary Figure 2.** Effect of biochar application and nitrogen fertilizer reduction on pH of floodwater

**Supplementary Figure 3**

**Supplementary Figure 3**. Effect of biochar application and N fertilizer reduction on soil pH. The BF, SF1, and SF2 refer to the basal, first and second supplementary fertilizer, respectively.
